# Improving primary care services for imprisoned women with severe mental illness (IP-SIS) Protocol Paper

**DOI:** 10.3310/nihropenres.13753.1

**Published:** 2025-02-28

**Authors:** Gloria Roden-Lui, Carolyn A. Chew-Graham, Jake Hard, Paula Harriott, Hannah King, Emma Mastrocola, Tammi Walker

**Affiliations:** 1 Department of Psychology, Durham University, Durham, UK; 2 School of Medicine, Keele University, Keele, England, UK; 3 Oxleas NHS Foundation Trust, Dartford, England, UK; 4 UNLOCK National Association of Ex-Offenders Ltd, Maidstone, England, UK; 5 Department of Sociology, Durham University, Durham, UK; 6 HMP Eastwood Park, Practice Plus Group, Reading, UK

**Keywords:** imprisoned women, severe mental illness, women’s prisons, primary health care services, health inequalities, racially minoritised women

## Abstract

**Background:**

A gap exists in the provision of care for imprisoned women with Severe Mental Illness (SMI), both in prison and on release to mainstream primary care. Women in such settings tend to have complex mental health problems, often with comorbid long-term physical health conditions (LTCs). These problems are compounded in women who are racially minoritised. The prison regime can be a barrier to addressing health needs of women: limited time out of cell and depletion of staff resources. Little is known about how imprisoned women with SMI use prison primary care services, to what extent services meet health care needs, and how services are experienced by different ethnic groups.

**Aims:**

1. To explore the range of primary care services delivered to imprisoned women with severe mental illness (SMI) in England and describe what is working well and the barriers to accessing care.
2. To develop a framework for use in women’s prison services to support the primary care of racially minoritised women with SMI.

**Methods:**

The proposed study comprises of three phases across female prisons in England. Purposive sampling will be used to capture different prison groupings.

- Phase 1: Semi-structured telephone/online interviews with prison primary care practitioners.
- Phase 2: Focus groups / one to one dicussions with imprisoned women with SMI, including women from a range of ethnic groups.
- Phase 3: Consensus groups with prison healthcare and non-clinical staff

**Patient and Public Involvement (PPIE):**

Co-applicant PH will be the PPIE lead, as a lived experience researcher, who supports engagement with imprisoned communities and PPIE in research. She will recruit and facilitate meetings with an ethnically diverse Lived Experience Advisory Group (LEAG), supporting members to participate in the Research Steering Group (RSG) that monitors study progress. She will be supported by Co-applicant HK who has expertise in supporting PPIE in engagement and participation in research.

**ISRCTN Registry:**

**ISRCTN10216673**

## Plain Language Summary

**Background and study aims:**

In the UK, approximately 5,000 women are in prison, at any one time, 71% of whom have mental health needs. Severe Mental Illness (SMI) includes mental health problems that can seriously impact someone’s day to day life. Examples include schizophrenia, bipolar disorder, personality disorder or complex emotional needs. However, SMI can also be defined as experiencing the symptoms of mental health problems without the formal diagnosis or having multiple ‘labels’ and diagnoses accumulated over time.

It is not known how imprisoned women with SMI access primary care services. We aim to understand more about the problems that imprisoned women with SMI might experience when attempting to use primary care services in prison and whether there are particular difficulties for racially minoritised women.

**We plan three phases:**

1. Interviews with practitioners who provide primary care services in women’s prisons.
2. Focus groups / one to one discussions with imprisoned women with a history of SMI.
3. Discussion groups with health and non-clinical prison staff with experience of working with imprisoned women with SMI.

**What will the results tell us?:**

This research will show the experiences of imprisoned women with SMIs and highlight different needs of women from different ethnic minorities. We will produce a framework to support the development of better services, supporting imprisoned women with SMI and on release to mainstream primary care services.

**Patient and Public Involvement and Engagement (PPIE):**

One of the co-applicants (PH) is the PPIE lead. They have lived experience of prisons and are experienced prison researcher. They will recruit members to the ethnically diverse Lived Experience Advisory Group (LEAG) and support them to participate as members of the study’s Research Steering Group (RSG). One of our other co-applicants (HK), who has expertise in supporting PPIE in engagement and participation in research, will support PH.

## Introduction

Imprisoned women comprise a small proportion of the total prison population in the UK, but many imprisoned women have high levels of health care need ( House of Commons Committee Report, 2022).

Approximately 5,000 women are incarcerated in the UK, 71% of whom have mental health care needs ( Forrester *et al.*, 2018; HM Inspectorate of Prisons, 2022). Imprisoned women often have disproportionately higher levels of SMI compared with men in prison, ( Ministry of Justice, 2013) and this percentage increased during COVID-19 ( Inquiry into Women’s Health in Prison: Briefing One, 2022). Severe Mental Illness (SMI) (such as psychosis and bipolar disorder) impacts on how women seek and interact with healthcare services within the prison setting.

Over the last decade, reports ( Corston, 2007; Home Office, 2007) and female offender strategy plans ( Ministry of Justice, 2018; Ministry of Justice, 2023) have advocated prison as a last resort for women. Community gender-based health disparities are amplified in prison, with women feeling more disempowered and facing increased challenges in accessing and receiving appropriate physical and mental health support ( Inquiry into Women’s Health in Prison: Briefing One, 2022). Racially minoritised women are more likely to be remanded or sentenced to custody, feel less safe in custody and have less access to mental health services ( Prison Reform Trust, 2021) Women’s experiences are compounded by complex needs arising from Adverse Childhood Experiences (ACEs), including trauma, abuse, separation from their children and family, homelessness, and unemployment ( Prison Reform Trust, 2021).

Comorbid physical conditions (such as cardiovascular disease and diabetes) in people with SMI leads to lower life expectancy and poorer quality of life ( Chesney *et al.*, 2014; Westhead *et al.*, 2023). People with SMI in England die on average 15 to 20 years earlier than the general population ( Public Health England, 2018b; Westhead *et al.*, 2023). There is often a focus on assessing and treating the mental health disorder to the detriment of identifying and managing physical health risks ( Lambert *et al.*, 2003). Living with SMI can disrupt the ability of women to engage with the primary care team because of challenging behaviours, impaired mental capacity, and women’s perceived lack of trust in care providers ( Public Health England, 2018b). SMI impacts on how imprisoned women can access secondary care outside the prison establishment, especially where there is additional security measures required ( Forrester *et al.*, 2018).

Medication used to treat women with SMI can contribute to poor physical health: anti-psychotics contribute to the development of obesity, hyperlipidaemia, diabetes, and heart disease, with increased morbidity and early death ( Public Health England, 2018a). Such long-term conditions, in the presence of SMI, can be a challenge to manage. These problems can be exacerbated for racially minoritized women, who may already be at greater risk of specific physical health problems such as diabetes, and will have different needs ( Patel *et al.*, 2017).

Aspects of the prison regime can be a barrier to addressing the health needs of women: low prison staffing levels, limited time out of cell, and the conflicts arising from addressing other urgent or unplanned events. Furthermore, some racially minoritized women are subject to additional difficulties, including language difficulties and some evidence of systemic racism in services ( Bansal *et al.*, 2022; Prison Reform Trust, 2017). The Royal College of General Practitioners [RCGP] guidance ( 2018, Chapter 1: The Definiton) states that people in prison should be ‘afforded provision of or access to appropriate services or treatment’, which are ‘at least consistent in range and quality...with that available to the wider community’. Healthcare in prison is guided by ‘equivalence of care’ ( Joint Prison Service, National Health Service Executive Working Group, 1999, p. 3). Yet barriers outside are amplified inside, including fear, anxiety, stigma, and logistical challenges including the need for escorts, long waiting times and disruption in the prison ( Hutchings & Davies, 2021). A decade of austerity, over-crowding, poor facilities, workforce instability, security constraints and poor data collection all affect the availability and accessibility of services. The report from the Royal College of Obstetricians and Gynaecologists (2020), discussing Racial Disparities in Women’s Healthcare shows that there is minimal evidence about their access to and experiences of healthcare in prison, leading to multiple calls for improved data about how race affects their access to health services.

A recent report, conducted by the All Party Parliamentary Group on Women in the Penal System, concluded that prisons “are unable to address the physical and mental health needs of women and in fact exacerbate them” ( Inquiry into Women’s Health in Prison: Briefing One, 2022, p. 1). Overall, there are no reliable reports about women with SMI, across different ethnicities, accessing primary care services in prisons and data on whether these services are adequately meeting their needs ( Walker *et al.*, 2021).

The need for research into how to provide acceptable care for under-served groups remains ( Bonevski *et al.*, 2014; Dowrick *et al.*, 2009). This study builds on work completed in 2020 called Physical Health Inequalities in Imprisoned Women with Severe Mental Illness: A plan for action, which established a group of multi-disciplinary academics, clinicians, charities, and individuals with lived experience who are keen to tackle primary care access for imprisoned women with SMI ( Walker *et al.*, 2021). This proposal aims to address the health inequalities and disparities experienced by women with SMI, including from racially minoritized groups, at risk of/with comorbid physical health problems in women's prisons. Furthermore, this study will contribute to a reduction in the current health inequalities and disparities experienced by imprisoned women with SMI. This research will also form a foundation for future work which will improve the primary health care services provided to women with SMI, including racially minoritized women, in prison and on release to mainstream primary care. This study will describe what is required in order to achieve equitable primary health outcomes for imprisoned women, despite all the barriers identified.

### Study aims

This study aims to explore the primary care services delivered to imprisoned women with SMI in England and describe what is working well and barriers to accessing care and to develop a framework for use in women’s prison services to support the primary care of racially minoritised women with SMI.

## Protocol

### Methods

***Design***

The proposed study has three phases and will be conducted over 18 months. Women’s prisons in England (none exist in Wales) will be invited to participate in the study. Up to six prisons will be selected to enable purposive recruitment of a diverse sample of participants. Purposive sampling will be used to ensure that the perspectives of women and staff from a range of different prison settings are included.

***Patient and Public Involvement and Engagement***

One of the co-applicants (PH) is the Patient and Public Involvement Engagement (PPIE) lead. They have lived experience of prisons and are an experienced prison researcher. They will be supported by another Co-I (HK), an experienced prison researcher. Together they will recruit the ethnically diverse Lived Experience Advisory Group (LEAG) and support members of the LEAG, including to participate as members of the study’s Research Steering Group (RSG).

The LEAG will meet every three months to discuss the progress of the study and contribute to data analysis. They will also support dissemination activities and be offered to contribute to papers in peer-reviewed journals, outputs directed at prisons and outputs directed for a lay audience.

The RSG will provide independent supervision and monitor the study, whilst providing clinical and professional advice where relevant. The RSG will have expertise from ethnically diverse researchers, academics and key stakeholders.

***Study participants***

Eligible participants will be aged over 18 years. Interpreters and translation services will be available if participants require assistance. The research team will be aware of the diversity and characteristics of participants within each prison when arranging and conducting interviews/focus groups/consensus groups. All members of the research team are experienced researchers in prisons, mental health, physical health, and other sensitive topics. They have prior experience and training to conduct research in prison settings.

*Phase 1: Semi-structured telephone/online interviews Primary Care Practitioners who work in female prisons.*

*Inclusion criteria*

- Aged 18+ years
- Qualified primary care practitioners who have worked in a female prison for at least 3 months.

*Exclusion criteria*

- Under the age of 18 years
- Qualified primary care practitioners who have not worked in a female prison for at least 3 months.

***Data collection***

 In Phase 1 of the study, telephone and online interviews will be conducted with primary care practitioners. This will allow the research team to conduct and generate rich data in a time-effective manner ( Miles & Huberman, 1994) as the team understands primary care practitioners have limited capacity and time in their workday. Approximately twenty-five participants from the six participating prisons will be recruited or until data saturation is achieved ( Saunders *et al.*, 2018). Recruitment of the primary care practitioners will be conducted by the research team via primary care practitioner forums and networks. Interested potential participants will be able to directly contact the research team. The research team will then provide an information sheet and at least 24 hours for potential participants to consider participating in the study. The research team will then return to the potential participants to answer any questions and obtain written informed consent from those willing to participate.

A topic guide will be used for the interview, which will have been developed by the research team and lived experience advisory group, drawing on existing literature about health disparities and inclusion. It will also explore current provision and the potential inequalities of these provisions for imprisoned women with SMI across different ethnic groups. In addition, the topic guide will also explore the preparation for imprisoned women on their transition back to mainstream primary care in the community. The interviews will last up to 60 minutes and will be transcribed verbatim, with consent.

***Data analysis***

The interviews will be digitally-recorded and transcribed verbatim, with any personal identifiable data removed from the transcript. The transcript will be reviewed by GR-L. NVivo 14 software (2023) will be used to store and manage the qualitative data collected in the interviews. Copyright license has been obtained via Durham University for NVivo 14 (2023). Alternative free open souce software that can be used includes, QualCoder, RQDA, Taguette etc. Data will be analysed using a framework approach ( Gale *et al.*, 2013). Framework analysis involves five steps of data management: familiarisation; constructing an initial thematic framework; indexing and sorting; reviewing data extracts; and data summary and display, followed by a process of abstraction and interpretation. The initial coding will be reviewed by the research team and final themes and framework agreed through discussion.

*Phase 2: Focus groups / one to one dicussions with Imprisoned Women with SMI from diverse ethnic backgrounds.*

*Inclusion criteria*

- Aged 18+ years
- Have the mental capacity to give informed consent (discussion through Safer Custody Team)

*Exclusion criteria*

- Under the age of 18 years
- Unable to provide informed consent.
- Pose a significant risk to self and/or others.

***Data Collection***

Potential participants will be invited to the study by the Safer Custody Team in each prison who will work closely with members of the research team. The Safer Custody Team, with the support of the primary healthcare team, will screen clinical/prison records to facilitate recruitment, for example participant eligibility and risk levels. Members on the research team have extensive experience for recruiting participants with potential capacity matters. Therefore, there will be a triangulation between the primary care team, prison staff, safer custody and the research members before identifying potential participants.

Approximately forty participants will be recruited across the six prisons to participate in focus groups / one to one dicussions. Imprisoned women with a history of SMI and detained for at least one month will be invited to participate. Purposive sampling will be used to recruit a range of imprisoned women from diverse backgrounds: Asian or Asian British, Black, or Black British, Mixed Heritage, Other Ethnic Groups and White. Eligible women will then be approached by the research team and invited to participate in a focus group. The research team will provide an information sheet and at least 24 hours for potential participants to consider participating in the study. The research team will then return to the potential participants to answer any questions and obtain written informed consent from those willing to participate. Participants will be asked basic demographic data such as age, gender, sexuality, ethnicity, and conviction status.

The topic guide for the focus group will be guided from the findings in Phase 1 and current evidence from literature. The questions used will be sensitive to language and cultural differences, with translation services available if required. Examples of topics that will be explored are what healthy means to them, barriers and facilitators to accessing primary care services whilst imprisoned, experiences of accessing services, experience of health care professionals, how prison primary care services could be improved, and how transition from prison primary care service to mainstream primary care can be facilitated upon release from prison. The focus groups / one to one dicussions will last up to 90 minutes and will be transcribed verbatim. Where participants prefer not to attend a focus group, the study team will offer individual hybrid method of data collection with participants such as one to one discussions or provide a written format for invidiyalsindividuals who wish to respond with this method. The research team understand that some individuals may find this method preferable to meet their needs and concerns and so the research team would be happy to provide this as an alternative.

***Data analysis***

All focus groups / one to one dicussions (n=6 focus groups) will be digitally recorded on an security cleared encrypted Dictaphone, see Data Management Plan below for more information. and transcribed verbatim, with any personal identifiable data removed from the transcript. Data analysis for Phase 2 will follow the same procedure as data analysis in Phase 1. For more information, see above Data Analysis Section in Phase 1.

*Phase 3: Consensus groups with Health and Non-Clinical Prison Staff in female prisons.*

*Inclusion criteria*

- Aged 18+ years
- Qualified or non-qualified prison staff who have worked in a female prison for at least 3 months.

*Exclusion criteria*

- Under the age of 18 years
- Qualified or non-qualified prison staff who have not worked in a female prison for at least 3 months.

***Data collection***

In Phase 3, the research team will recruit participants. Approximately, sixty health and non-clinical prison staff will be recruited to participate in six online consensus groups, held one in each of the six prisons. The research team will recruit individuals of different ethnicities. Recruitment of participants will be conducted by the research team in person at each of the six prisons and also via email. The research team will provide an information sheet and at least 24 hours for potential participants to consider participating in the study. The research team will then return to the potential participants to answer any questions and obtain written informed consent from those willing to participate.

The aim of the Phase 3 is to develop a culturally and racially sensitive framework to understand the barriers and facilitators to providing primary care for women with SMI both within prison and following release to transition to mainstream primary care. Findings from the literature, and from Phases 1 and 2 of the study will be presented in the consensus group. Participants will then discuss the findings to develop recommendations for prison staff training, policy and standards, and transition plans to mainstream primary care for imprisoned women. The consensus groups will be conducted online, recorded, and last up to 90 minutes.

***Data analysis***

The consensus groups will be recorded and transcribed verbatim, with any personal identifiable data removed from the transcript. Data analysis for Phase 3 will follow the same procedure as data analysis in Phase 1 and Phase 2. For more information, see above Data Analysis Section in Phase 1.

***Data management plan***

All participants will be provided with an information sheet when recruited to participate in the study and offered at least 24 hours to consider whether they wish to participate. Once participants agree to participation, they will be provided with a written consent form. After the interview/focus group, participants will be provided with a debrief sheet and signposting for support if the content of the interview/focus group affected them negatively. The debrief sheet also provides contact details for complaints and other issues that may arise after the study.

Participants can withdraw from the study at all times, without giving a reason, but the research team will keep information about them that they already have. No-one outside of the study team will be informed of the withdrawal.

Once data analysis has started,the research team will anonymise all the data, meaning that they will not be able to let participants see or change the data they hold about them. This is because the research team will no longer be able to match the participant to their data, due to the data being anonymised. By anonymising the data, this ensures confidentiality for the participants. Their confidentiality will be maintained at all times unless there is a risk to self or others and then disclosure to the relevant authorities is necessary. This will be clearly explained in the consent form.

The encryted Dictaphone that will be used to digitally record focus groups and interviews will be pre-authorised by the prison service. Once the recordings have been transcribed, the recordings on the Dictaphone will be deleted. Online interviews will be recorded using Microsoft Teams and again, once recordings have been transcribed, the recordings will be deleted. All participants will be asked to consent to be recorded in the written consent forms. Data collected will be completely anonymous. The data will be stored securely on an electronic drive at the Durham University, and the Principle Investigator (PI) will be responsible for the data. Data/quotes published will be anonymised. In accordance with university policy, research data will be kept for 10 years. Participant identifiable data will be kept separate to anonymised data, stored in a secure locked cabinet with the PI. No one outside the research team will have access to it and the participant identifiable data will then be permanently destroyed after the research period. All data will be stored in line with General Data Protection Regulation ( GDPR, 2016) guidelines and researchers managing data will also be bounded by GDPR regulations and confidential clauses in contracts.

***Patient and Public Involvement and Engagement***

One of the co-applicants (PH) is the Patient and Public Involvement Engagement (PPIE) lead. They have lived experience of prisons and are an experienced prison researcher. They will be supported by another Co-I (HK), an experienced prison researcher. Together they will recruit the ethnically diverse Lived Experience Advisory Group (LEAG) and support members of the LEAG, including to participate as members of the study’s Research Steering Group (RSG).

The LEAG will meet every three months to discuss the progress of the study and contribute to data analysis. They will also support dissemination activities and be offered to contribute to papers in peer-reviewed journals, outputs directed at prisons and outputs directed for a lay audience.

The Research Steering Group will provide independent supervision and monitor the study, whilst providing clinical and professional advice where relevant. The RSG will have expertise from ethnically diverse researchers, academics and key stakeholders.

***Dissemination***

All dissemination will be informed by the LEAG and the outputs will be co-produced. The research team will also host knowledge cafes and invite professionals, charities and third sector organisations who are interested in this topic, to participate and connect with each other. The event will have a scriber illustrating the key messages and ideas of the event, which will later be shared. In addition, the research team will provide support to producing a podcast with the National Prison Radio and submit an article for Inside Time, discussing the findings of the study. There will also be planned academic publications, and presentations at academic and clinical conferences.

***Reflexivity***

Interviews, focus groups and consensus groups will be conducted by GR-L, PH, HK and supervised by TW and CC-G. The research team has an extensive background in prison and other complex forensic settings. The research team are all trained in qualitative data collection with vulnerable individuals. Regular supervision and debriefs will be held to ensure researcher safety. Any prior personal or working relationships with participants will be disclosed prior to data collection.

## Discussion

Imprisoned women have needs that relate to mental and physical ill-health, drug and alcohol dependence and self-harm ( Public Health England, 2018a). Despite this, women, some with complex mental health needs, may be (inappropriately) remanded to prison as a place of safety by the courts, often due to the lack of alternative mental health services in the community ( Inquiry into Women’s Health in Prison: Briefing One, 2022). Thus, Davies *et al.* conclude that 'It is important to understand how gender, ethnicity and position in the criminal justice system impact on access to and the use of health care services' ( Davies *et al.*, 2022, p. 6).

Minimal evidence exists about how imprisoned women with SMI use primary care services, to what extent services are meeting their health care needs, and how these services are experienced by different racial groups ( McCann *et al.*, 2019; Prison Reform Trust, 2021; Inquiry into Women’s Health in Prison: Briefing One, 2022). Our research will be an important first step at addressing these gaps in knowledge by developing a framework that can be applied and evaluated. A recent inquiry into women’s health and wellbeing in prisons ( Inquiry into Women’s health in prisons: Briefing One, 2022), concluded that the prison environment is itself damaging to women’s mental health, compounds their victimisation (the majority of whom have experienced violence or abuse prior to prison) and exacerbates health inequalities for racially minoritized women.

### Strengths and limitation of the study

A key strength of this study is the patient and public involvement and engagement. The co-applicant with lived experience and the LEAG will have a significant influence at all stages of this research. This will allow the research to be sensitive to the lived experience of the participants and allow in-depth insight to be collected in the interviews and focus groups. The research team also have a diverse background in prison, imprisoned women, mental health, and physical health research, allowing and exploring interdisciplinary ideas on interpreting the data.

A limitation to this study is the recruitment and sampling of the imprisoned women with SMI. There are restrictions placed on the research team, as with all prison research, due to the complex nature of the setting. Therefore, the research team will only be able to recruit participants who are not considered high risk to themselves and/or others, and participants who the Safer Custody Team have screened as eligible. Thus, the data may only reflect a subsection of the population of imprisoned women with SMI.

Our research will inform service planning and address health inequalities by illustrating the experiences of imprisoned women with SMIs. It will identify their intersectional and differential needs, including those of racially minoritised women, and develop a culturally and racially sensitive framework for prison primary care services. This research, conducted in a sensitive way with women’s voices at the fore, will lead to improved engagement with health services and ultimately better health outcomes for imprisoned women.

## Ethics and consent

Ethical approval for this study has been submitted to the Seasonal Review Ethics Committee (REC; IRAS 342813; 22nd August 2024) with conditions stating that once HM Prison and Probation Services National Research Committee (HMPPS NRC) has been provided a Health Research Authority (HRA) approval can be provided. The study is currently awaiting approval from the HMPPS National Research Committee (NRC).

The researcher will go through the participant’s information sheet and address any questions that arise. Time will be allowed if the potential participant more time to consider their involvement in the study. The research team will obtain written informed consent from all participants. The research team hereby declare that they adhere to the Declaration of Helsinki (1964).

## Data Availability

No data are associated with this article.
