# Supplementary material for: Improving primary care services for imprisoned women with severe mental illness (IP-SIS) Protocol Paper
Source: NIHR Open Res. 2025 Feb 28;5:14. [Version 1] doi: 10.3310/nihropenres.13753.1 (PMC12489449; doi:10.3310/nihropenres.13753.1)
Supplement: Peer review history

# Reviewer report: Reviewer response for version 1

**DOI:** 10.3310/nihropenres.14940.r35035

**Published:** 2025-05-10

**Authors:** Jack Tomlin

**Affiliations:** 1 University of Greenwich, Greenwich, UK

Thank you very much for this chance to review this work. I think it is an important topic and worthy of study. Such research in prisons is hard to conduct and takes a lot of prep and administration - kudos to the research team for this. My thoughts:

 I think the literature review could offer a more extensive summary of the literature on people in prison's experiences/attitudes of prison health care to see what broader lessons could inform this work. 

- Please define 'challenging behaviours' and state these instead?
- Please d SMI in intro.
- Re-read for typos e.g., 'especially where there is additional security measures required'
- Please comment on social/structural determinants of health and situate this work within a model/framework of this?
- Clarify why up to 6 prisons included?
- How define 'diverse sample' and how this will be 'achieved'?
- How the authors drawn on NIHR guidance to recruit ethnically diverse LEAG? If so, could this be mentioned? there is lots of developing good practice in this area. 
- What activities will the LEAG do (i.e., in more detail than 'meet to discuss')? 3 months is a reasonably lengthy gap between meetings, how will the team ensure/maintain engagement? What level of research involvement will the LEAG members be operating on/able to choose?
- Data saturation is a grounded theory concept - will this be a GT study? Maybe 'information power' is more helpful here?
- Please comment on the strengths/weaknesses and implications of mixing FGs and individual interviews in relation to the kind of data collected/co-constructed.
- Will interviews in phase 2 explicitly address facilitators/barriers for women who are minoritized? Or will their answers be analysed separately? How will the answers to this research focus be teased out?
- Is the following section included twice? 'Patient and Public Involvement and Engagement'
- Can the authors engage more extensively with reflexivity? How do they anticipate their biographical, experiential and political leanings/selves will influence the conduct and analysis of the work? or their choice to conduct this project? 
- Will this protocol need to change if the ethical review process requests methodological changes?
- Please make clear why the authors will not develop not a framework for all women, with specific modules/features etc for racially minoritized women? Why investigate the experiences of women/females more broadly and then only develop framework for certain group(s)? I think this output is needed/valuable, but could be justified more explicitly.

Is the study design appropriate for the research question?

Yes

Is the rationale for, and objectives of, the study clearly described?

Yes

Are sufficient details of the methods provided to allow replication by others?

Partly

Are the datasets clearly presented in a useable and accessible format?

Not applicable

Reviewer Expertise:

Forensic mental health

I confirm that I have read this submission and believe that I have an appropriate level of expertise to confirm that it is of an acceptable scientific standard, however I have significant reservations, as outlined above.

# Reviewer report: Reviewer response for version 1

**DOI:** 10.3310/nihropenres.14940.r35030

**Published:** 2025-05-08

**Authors:** Laura Caulfield

**Affiliations:** 1 University of Wolverhampton, Wolverhampton, UK

The protocol outlines a much needed area of study and action. Healthcare in prisons can fall far short of acceptable standards, and this can be particularly so in the women’s estate where there are high levels of need. I am reassured to see the focus on women from a variety of ethnic groups, given emerging evidence that Black women in particular experience worse treatment (health and otherwise) than other groups in prison.

 The study design, including the perspectives of practitioners and women in prison, is appropriate – as is the final consensus phase. I would encourage the team, perhaps through the LEAG, to consider the ethnic diversity of the practitioner participants. In my experience ensuring diversity in all participant groups can bring different insights.

 Under dissemination there is no mention of routes into formal healthcare and justice fora, which seem quite crucial for such important research. 

 There are one or two minor typos that should be addressed |(here are the ones I spotted):

 A missing full-stop

 invidiyalsindividuals 

 Principle Investigator – Principal Investigator

 Occasional use of US spelling (e.g. z rather than s)

Is the study design appropriate for the research question?

Yes

Is the rationale for, and objectives of, the study clearly described?

Yes

Are sufficient details of the methods provided to allow replication by others?

Yes

Are the datasets clearly presented in a useable and accessible format?

Not applicable

Reviewer Expertise:

Prisons research, women in criminal justice, qualitative and mixed-methods research.

I confirm that I have read this submission and believe that I have an appropriate level of expertise to confirm that it is of an acceptable scientific standard.

# Reviewer report: Reviewer response for version 1

**DOI:** 10.3310/nihropenres.14940.r35038

**Published:** 2025-04-14

**Authors:** Emily Glorney

**Affiliations:** 1 Royal Holloway University of London, Egham, England, UK

The protocol sets out intentions to understand and propose suggestions for improving access to, use and experience of primary care services among women in prison. This is an under-researched area and the work has potential for improving patient services.

 There are a few areas of the protocol that would benefit from elaboration.

 It would be helpful if severe mental illness were clearly operationalised for the purpose of this study. The plain language summary makes reference to personality disorder but this is not included in descriptions elsewhere in the main body of the protocol. The definition is important because this links to participant inclusion criteria and experience of access to and use of primary care services relating to psychological wellbeing in prisons. This also raises a question about co-morbidity of severe mental illness and personality disorder among women in prison (the complexity of need has been acknowledged in the protocol). If there is a focus specifically on severe mental illness (e.g. psychosis, bi-polar disorder) then how will the research team be confident that findings are specific to women with related needs; or how might co-morbidity with personality disorder be accounted for?

 The penultimate paragraph of the Introduction suggests that the research will explore primary care service use and access by women in prison with a severe mental illness. This will include an exploration of differences in use, access and experience across race and ethnicity, to support equity of access for racially minoritised women. However, in the Study Aims section, the emphasis seems to be on an explicit investigation of barriers and facilitators to accessing primary health care among racially minoritised women. The study aims require clarification.

 It is unclear how the Aim to “explore the range of primary care services delivered to imprisoned women with SMI” (page 1) will be addressed. Further information is required.

 Information about the number of women’s prisons in England would be useful, to contextualise the intention to sample from a maximum of six; why might that be the case? Are there a total of six women’s prisons in England?

 Do the inclusion criteria for Phase 2 require for participants to have a diagnosis of a severe mental illness?

 How will the research team ensure that there is racial and ethnic diversity in the sampling of staff (Phases 1 and 3)? Or how will the research team account for possible lack of diversity in the findings and proposed recommendations?  There might be an interface here with the LEAG. There is reference to targeted recruitment in the description of Phase 3 but this point requires elaboration.

Is the study design appropriate for the research question?

Partly

Is the rationale for, and objectives of, the study clearly described?

Partly

Are sufficient details of the methods provided to allow replication by others?

Partly

Are the datasets clearly presented in a useable and accessible format?

Not applicable

Reviewer Expertise:

Forensic mental health; qualitative research

I confirm that I have read this submission and believe that I have an appropriate level of expertise to confirm that it is of an acceptable scientific standard, however I have significant reservations, as outlined above.

# Reviewer report: Reviewer response for version 1

**DOI:** 10.3310/nihropenres.14940.r34848

**Published:** 2025-03-26

**Authors:** Roland Jones

**Affiliations:** 1 University of Toronto, Toronto, Canada

The authors are to be congratulated for undertaking a study focusing on an often overlooked and underserved demographic. Incarcerated women represent a minority within the prison population, yet experience disproportionately high levels of physical, psychological, and social health needs. It is therefore essential that health services are tailored to address the specific challenges faced by this group.

 The proposed three-phase, 18-month study aims to investigate current primary care practices in women’s prisons in England, with a view to identifying components of good practice. The involvement of a co-investigator with lived experience is a particular strength and aligns well with current priorities for inclusive, patient-centred research.

 To strengthen the protocol, the following points may be considered:

1. The stated aim to “explore the primary care services delivered” would benefit from greater specificity. It would be helpful to clarify what aspects are being examined—e.g., access, quality, continuity, responsiveness to mental health needs, or cultural competence—so that the study focus is clearly defined.
2. The aim refers to racially minoritized women with serious mental illness as the target population. This is important and commendable. However, while the methods mention purposive recruitment from a range of diverse backgrounds, it is unclear how central ethnicity is to the study design. Further clarification on whether the study is focused primarily on racially minoritized women, or on all women with SMI with attention to ethnic disparities, would be useful.
3. The protocol indicates that “up to six prisons” will be included. It would be helpful to specify the minimum number of prisons and the anticipated number of participants per phase, to better assess feasibility and generalizability. More detail is also needed on the purposive sampling approach—for example, the specific criteria guiding participant selection and how this will be operationalized in practice.
4. The protocol notes that 60 staff will be recruited to participate in six online consensus groups, each lasting up to 90 minutes. It would be helpful to clarify the format—for example, whether this will involve six separate groups of 10 individuals meeting once, or whether participants will engage in multiple sessions. If the groups meet separately, the authors should describe the process by which consensus will be reached across all participants (e.g., through thematic synthesis, voting rounds, or a Delphi-style approach).

Overall, this is a well-conceived and much-needed study. Addressing these points would further strengthen the clarity of the protocol.

Is the study design appropriate for the research question?

Partly

Is the rationale for, and objectives of, the study clearly described?

Partly

Are sufficient details of the methods provided to allow replication by others?

Partly

Are the datasets clearly presented in a useable and accessible format?

Not applicable

Reviewer Expertise:

Prison psychiatry, forensic mental health, epidemiology.

I confirm that I have read this submission and believe that I have an appropriate level of expertise to confirm that it is of an acceptable scientific standard, however I have significant reservations, as outlined above.
